# The meanings attributed by obstetricians to planned home birth

**DOI:** 10.1590/0034-7167-2024-0434

**Published:** 2025-11-07

**Authors:** Laiane Ribeiro Viana, Diego Pereira Rodrigues, Valdecyr Herdy Alves, Bianca Dargam Gomes Vieira, Ediane de Andrade Ferreira, Giovanna Rosario Soanno Marchiori, Márcia Vieira dos Santos, Luana Asturiano da Silva

**Affiliations:** I Universidade Federal do Pará. Belém, Pará, Brazil; II Universidade Federal Fluminense. Niterói, Rio de Janeiro, Brazil; III Universidade Federal do Amapá. Macapá, Amapá, Brazil; IV Universidade Federal de Roraima. Boa Vista, Roraima, Brazil; V Hospital Servidores do Estado. Rio de Janeiro, Rio de Janeiro, Brazil

**Keywords:** Home Childbirth, Physicians, Obstetricians, Humanizing Delivery, Health Policy., Parto Domiciliario, Médicos, Obstetras, Parto Humanizado, Política de Salud.

## Abstract

**Objectives:**

to understand the meanings attributed by obstetricians to planned home birth.

**Methods:**

this is a qualitative study carried out with 13 obstetricians from a regional hospital in Belém, Pará, recruited using the snowball sampling technique. Semi-structured interviews were used, recorded and transcribed in full to perform content analysis with the support of ATLAS ti 22.7 software.

**Results:**

there was a lack of sizing, support maternity wards and high purchasing power to guarantee childbirth. In addition to the lack of structure and professionals, the lack of knowledge on the subject and public policies constitutes an obstacle in the Brazilian health sector.

**Final Considerations:**

it is understood that, due to the meanings attributed by obstetricians, home birth constitutes a risk factor, especially due to the lack of structure and support in the Brazilian health system.

## RESUMEN

**Objetivos:**

comprender los significados atribuidos por los obstetras al parto domiciliario planificado.

**Métodos:**

se trata de un estudio cualitativo, realizado con 13 obstetras de un hospital regional de Belém, Pará, reclutados mediante la técnica de muestreo en bola de nieve. Se utilizó una entrevista semiestructurada, grabada y transcrita íntegramente para realizar el análisis de contenido con apoyo del software ATLAS ti 22.7.

**Resultados:**

faltó dimensionamiento, apoyo a las maternidades y alto poder adquisitivo para garantizar el parto. Además de la falta de estructura y profesionales, la falta de conocimiento sobre el tema y las políticas públicas constituye un obstáculo en el contexto de la salud brasileña.

**Consideraciones Finales:**

se entiende que, a partir de los significados atribuidos por los obstetras, el parto domiciliario constituye un factor de riesgo, especialmente por la falta de estructura y apoyo en el sistema de salud brasileño.

## INTRODUCTION

Until the beginning of the 19^th^ century, there was a predominance of childbirth assistance by midwives. But this reality changed drastically with the creation of hospitals, promoting a banner of greater safety and reduction of perinatal mortality^(1)^. Therefore, the birthing process underwent drastic changes, which can sometimes be seen as a reduction in women’s autonomy over choices regarding how to conduct this entire moment, which should have a focus on the decisions of women giving birth.

This obstetric overview exerts intense interventions on the female body, such as medicalization, episiotomy, Kristeller maneuver, cesarean section and other introduced practices, and sometimes, they are used without any verifiable clinical justification^(2,3)^, sometimes compromising pregnant women’s entire personal planning regarding birth.

With this effect, in the mid-21^st^ century, with the popularization of the internet and the spread of information, there was a sharing of experiences and exchanges within the women’s movement, seeking alternatives to the abuses committed in healthcare institutions. Planned home birth (PHB) emerged to break with the biomedical and interventionist model, ensuring better care and respect for women.

The scope of birth in Brazil is portrayed with a predominance of hospital births, with less than 2.4% of home births^(4)^. In the state of Pará, there is also this similarity, but with a difference in reality, as home births in the Amazon region are cultural, especially in places with limited infrastructure, which often have a traditional midwife, whether in communities with traditional populations, such as riverside communities, *quilombolas* or indigenous peoples. Culturally, there is a lack of urban centers and care units, and thus, traditional midwives’ practice is the sole responsibility of women in the area of childbirth^(5)^. Another fact is that the state does not have data on teams specialized in home births, such as physicians and nurses, and there are initiatives by a few private teams, which not all women have the financial means to hire.

Thus, we can highlight that, in several countries, women give birth through PHB, such as the United Kingdom, Canada, Australia, the Netherlands, Sweden, New Zealand, among others. Assistance is shown to be as safe as in births in hospital units^(6)^. Two studies highlight the safety of low-risk home births assisted by nurses-midwives in areas where this modality is integrated into the health system^(7)^. Thus, no differences in perinatal/neonatal mortality were found in their cohort study of 11,493 births vs. 11,493 hospital births in Ontario, Canada. A meta-analysis concluded that evidence on maternal outcomes consistently supports PHB in these conditions^(8)^.

Thus, there is an increasing demand for a model that breaks with traditional services, with rigid institutional norms, and that promotes more individualized assistance, with qualified listening and trust in the real needs of women and their families, making the choice of place of birth an informed, conscious and intuitive decision^(9)^. In this regard, the World Health Organization (WHO), the American College of Obstetricians and Gynecologists (ACOG) and the National Institute for Health and Care Excellence (NICE) reaffirm the safety of PHB, with fewer interventions and fewer associations of lacerations, infections and morbidity^(8)^. In this way, they encourage and recommend that women at low risk give birth through this experience, ensuring greater respect and autonomy^(10-13)^.

However, in the Brazilian scenario, the Ministry of Health (MoH), through Technical Note 2/2021 of the Department of Strategic Programmatic Actions of the Secretariat of Primary Healthcare, weakens women’s autonomy regarding the choice of home birth, as well as healthcare professionals’ work, such as nurses-midwives. The aforementioned technical note reinforces the hegemonic obstetric model centered on medical, hospital-centric and technocratic knowledge, which states that there are no large randomized clinical studies, with rigorous methodology, that support the safety of birth in a home environment, thus advising against the choice of home birth as a place of birth^(14)^.

It is necessary to reaffirm that this technical note is aligned with other measures taken by the federal government (2019-2022), such as the new Pregnant Women’s Handbook, good practices based on scientific evidence, and the Maternal and Child Healthcare Network, which are contrary to women’s rights, as well as non-medical professionals. This fact is based on a cooperative stance in medicine that directly affects women’s power to choose the Brazilian government. With the new progressive government, all measures that created obstacles to reproductive health and also to other healthcare professionals’ work were revoked. Currently, the Brazilian government has established the Alyne Network in support of the 2030 Agenda Sustainable Development Goals with maternal mortality and breaking all setbacks in policies and initiatives for women’s health.

Thus, the Brazilian government’s technical note in 2021 propagates the hegemonic model of obstetric care and does not guarantee PHB, even though recognized institutions, such as the WHO, ACOG and NICE, have recommended such implementation. Another fact is that the Brazilian National Supplementary Health Agency does not guarantee, in the list of procedures, PHB, which shows the total disconnection of MoH with international recommendations and scientific evidence, with only the maintenance of the care model.

Even so, it is necessary to understand the meanings of obstetricians regarding their perception of PHB, since women may enter obstetric network due to complications resulting from care, and their meanings demonstrate an understanding of the core of this phenomenon, even though this care is not directly interconnected to the obstetric care network. Thus, the study has as its guiding question: what are the meanings attributed by obstetricians working in hospital units about PHB?

## OBJECTIVES

To understand the meanings attributed by obstetricians to planned home birth.

## METHODS

### Ethical aspects

The study was approved by the Research Ethics Committee of the *Universidade Federal do Pará* Institute of Health Sciences, as provided for in Resolution 466/2012 of the Brazilian National Health Council. To preserve confidentiality, anonymity, and reliability, deponents were identified by the letters “OB” for obstetrician, followed by a numerical number corresponding to the sequence in which the interviews were conducted (OB1, OB2, OB3, …, OB13), in addition to guaranteeing voluntary participation by signing the Informed Consent Form.

### Study design

This is a qualitative, descriptive, exploratory study. Participants were recruited using the snowball sampling technique^(15)^. This technique is used with the nomination of seeds by each interviewee, with the indication by them, with the production of new contacts from the recruitment process of participants^(15)^. To achieve methodological rigor, the COnsolidated criteria for REporting Qualitative research (COREQ) instrument was used, which constituted the tool for the development of this qualitative research.

### Participants and data sources

The study was conducted with 13 obstetricians working at the *Hospital Regional Abelardo Santos*, located in the city of Belém, Pará, Brazil. The health unit is a public service in the state of Pará, and is one of the main hospitals in the Metropolitan Region for obstetric care in low-risk pregnancies.

The onboarding process began with introducing the study to the unit’s coordinators. Bearing this in mind, the coordinators were asked to provide three physicians’ contact information. Once they had this contact information, initial interaction with them began via WhatsApp^®^ application, explaining the study’s topics, objectives, data collection techniques, risks, and benefits. After this process, inclusion criteria were being an obstetrician and working in the obstetric center. Exclusion criteria were being healthcare professionals from other health areas. It should be noted that no participants were excluded from the study.

After this application, interviews with each participant were scheduled at the unit. After the interview, participants provided three more contacts to carry out the recruitment process, according to the snowball sampling technique, until the number of study participants was established.

Data collection was completed and the number of 13 study participants was established through the process of theoretical saturation^(16)^, which refers to when the data do not provide new elements to guide or deepen the theorizing^(16)^, thus providing a core of meaning about the problem. Thus, when the number of ten participants was reached, it turned out that the data were not providing any new elements, and thus three more participants were established, succeeding the determined number of study participants.

### Data collection and organization

The semi-structured interview with open-ended and closed-ended questions was used as the data collection technique, conducted from November 2023 to March 2024, which lasted an average of 45 minutes. The interviews took place individually, in a single moment, in a reserved room in the hospital unit, with only the participation of the main researcher and the interviewee, to be carried out by only one interviewer during the data collection technique application.

The semi-structured interview contained information on socioeconomic, educational and professional data, and two triggering questions, namely: what is your perception of PHB in the Brazilian context? What are the main obstacles to accessing and assisting PHB? Each interview used an MP3 recording resource to capture the testimony, with prior authorization of participants.

Statements were transcribed in full. After this stage, they were validated by the interviewees, showing at another time the sending of their transcribed speeches via WhatsApp^®^, thus validating the data, as determined by COREQ.

It is worth noting that a pilot study was carried out with three participants, who were not included in the study, which aimed to verify the interview’s suitability with the research objectives and physicians’ statements.

Data collection was conducted by only one researcher, demonstrating mastery of the technique used, thus avoiding different procedures, while the research team will be responsible for processing and analyzing the data.

### Data analysis

Content analysis^(17)^ was performed in data organization and treatment. First, the first stage, pre-analysis, was carried out, with a quick reading and initial contact with the data, seeking the first message obtained, with the selection of documents that will compose the study *corpus*, according to data on exhaustiveness, representativeness, homogeneity and relevance^(17)^.

Then, for material exploration^(17)^, coding interventions were constructed. Thus, the functionality of ATLAS.ti 22.7 software was submitted to inductive analysis, considering the coding of segments of statements with code assimilation and composition conception, namely: obstetric risk; insufficient structure; lack of trained professionals; high purchasing power; lack of information; public policies; and deficiency of professional teams, with the purpose of searching for the unity of meaning from the codes that emerged from the process.

In the last chronological pole of the analytical process, treatment of results, inference and interpretation, constructive elements were classified by the process of mutual exclusion, such as homogeneity, relevance, objectivity, fidelity and productivity, aiming at categorization^(17)^. In this last stage of the inductive analysis procedure, the collected contents were submitted, each one linked to the term documents, with the acronym accompanied by numbering, as appropriate for the software, starting from D1 to D15. Subsequently, the citations of components of the documents were coined in such a way that they were listed with codes, which constituted composition names with the meaning of the citation decoded by the researcher.

From this stage onwards, imperative codes were counted, combined with the inductive topics that emerged predominantly in the interviews and, subsequently, the saturation of these codes, through the recurrence of meanings, expressing the concretization of a dictionary of codes. These allow the design of the set of codes and the corresponding citations, through assimilation, thus, of the units of meaning, with the classification of constructive information and the regrouping of meanings, which arise from the conjunction of participants’ returns, motivating the construction of categories^(17)^.

Through this process, the following categories were constructed: 1) The meanings of planned home birth in Brazil; 2) Challenges and realities of access to planned home birth in the Brazilian context; 3) Risk and safety in planned home birth. The discussion was based on public policies in the field of labor and birth and scientific literature on PHB.

## RESULTS

The 13 interviews conducted revealed various demographic, educational and professional characteristics of participants. The distribution by gender shows that ten are female and three are male. Concerning age, seven are between 20 and 30 years old, four are between 40 and 50 years old, and two are over 50 years old.

In relation to race/ethnicity, six self-identified as white, six as brown, and one as black. Religious affiliations varied, with five Catholics, two Evangelicals, two without religion, two Christians (without specifying denomination), one Spiritist, and one without specified religion. Regarding marital status, six were single, six were married, and one was divorced.

All have a medical degree, eight from public institutions and five from private institutions. The training institutions include the *Centro Universitário do Pará, Universidade Federal do Pará, Centro Universitário Unifamaz* and *Universidade Estadual do Pará*.

In the field of specializations, everyone had specializations. None of the interviewees had a *stricto sensu* graduate course.

### The meanings of planned home birth in Brazil

According to the meanings attributed by obstetricians, they mention the risk inherent to PHB, such as hemorrhage, dystocia and unforeseen complications. Thus, it is shown that, based on their meanings, a negative aspect by physicians, especially with the lack of immediate support in emergency cases, is worrying, according to statements:

*Look, I think it’s dangerous, because ideally the patient should be in the hospital if she has puerperal hemorrhage, which is one of the main complications of normal birth, so I think it’s dangerous, because then she won’t have the assistance, she needs at the time, if she has a postpartum complication.* (OB1)

*Look, I want to tell you, I don’t have a good conception, acceptance, let’s say, of home birth due to the risks, you know? Because it’s an unknown. At that time, everything could go well, but things could go wrong, you put the life of a woman and a child at risk* [...] *suddenly, the birth goes well, and then the placenta gets stuck and causes hemorrhage, and it puts her at risk and you can’t! It’s a matter of a short time before you have to go from your home to a hospital.* (OB2)

*But it can also get complicated and require hospital care and everything else. I think it’s risky in Brazil, in the context of Brazil, considering our health system. In general, I think our training is risky. There’s a higher risk of complications during the baby’s birth.* (OB4)

PHB encounters barriers, according to the witnesses, such as the lack of adequate infrastructure for home births in the Brazilian context, especially due to the lack of hospital units, scarce equipment and staffing, professionals trained in maternity emergencies, according to the following statements:

*There would have to be a backup hospital. If there was a backup hospital close to the home locations, that could contribute in a positive way, right? If she has a home birth, if there are any complications, she can go to the nearby hospital for help.* (OB1)

*There is no medical support normally, you know?* [...] *if there is no hospital nearby and no urgent and emergency obstetric care, I think it is dangerous... most birthing centers still do not have a medical professional on call, which is sad.* (OB3)

*A puerperal hemorrhage that you have to act on immediately, right? It’s difficult to act on a home if you don’t have an ambulance there to quickly take her to the hospital.* (OB6)

*Home birth is a distant reality in the SUS principles, because everything starts in the basic unit, and the service is precarious and there is no assistance.* (OB10)

In terms of staffing, there is a need for professionals with experience and prepared to conduct home births. Thus, the interviewees frequently mention the comparison with other countries, where there is concern and support for professionals, as shown in the following statements:

*Dangerous, there is no medical support normally, you know?* [...] *even in England, which has nurses-midwives trained for normal birth, they always have a physician on call, always, that is not our reality here in Brazil yet, unfortunately, not yet, you know?* [...] *most birthing centers still do not have a medical professional on call, which is sad, you know?* [...] *not that obstetric nursing is not capable of undoing, for example, an incident during vaginal birth, but I think that, in the home environment, it is an unnecessary risk.* (OB3)

*It is necessary, in fact, that the professionals in charge are quite experienced. I think a team is necessary, because home birth can be very successful, it is not if* [...] *but it can also be complicated and require hospital care and everything else* [...] *in Brazil, in the Brazilian context, aiming, therefore, at our health system, generally our training, I think it is risky.* (OB4)

*The person who acts as a doula, in the case of a home birth, has, in addition to experience, the knowledge to know how far she can go.* (OB8)

The meanings attributed by deponents show that PHB is more viable for women with high purchasing power, which does not reflect the reality of the population assisted by the SUS, as the following statements show:

*When I think about labor planned to happen at home, I am thinking about people who have money to be able to have everything; I am not thinking about the person who is assisted by the SUS.* (OB7)

*Many people don’t know, they only have a basic understanding of what prenatal care involves, it’s not talked about yet. Investments are also precarious, even people’s homes are sometimes precarious, you know the reality of our country, so they end up coming to the hospital where they feel safe in case of complications.* (OB11)

In this way, the interviewees show, based on their perception, the negative meanings regarding PHB in the scope of obstetric care in the Brazilian context.

### Challenges and realities of access to planned home birth in the Brazilian context

It is important to mention that the statements compare rural and urban areas, i.e., in areas far from cities, there is greater access to home births due to the lack of resources and infrastructure, which can pose high risks to the mother and baby. Furthermore, in large cities, easy access to hospitals makes hospital birth the predominant choice:

*I think it’s easier in regions that don’t have hospitals, like the countryside, where there are many midwives. I think access should be easier in these places. In big cities, I think it’s easier to go to the hospital.* (OB1)

*I don’t think access is very encouraged. I don’t see it that way, at least not here in our region, right? Home births are not encouraged. Even though people work with this, there are even groups for natural births, but they are always carried out inside the hospital.* (OB4)

*Precarious because there is no support, nor is prenatal care provided properly. So, I see that there is no access. We see it more in the countryside, but there is no access there and it is difficult for them to travel, and they do not even know what a planned home birth is, because no one talks about it, no one promotes it, not even in prenatal care, so I say again that everything starts with primary care.* (OB10)

The lack of knowledge and adequate dissemination by Basic Health Units linked to PHB and the existence of taboos surrounding this topic constitutes a barrier. This means that many women are not informed about the possibility of a planned home birth, as per the statements:

*There is still a certain taboo. I think that pregnant women themselves do not know about home birth.* (OB5)

*Precarious because there is no support, but access is what they have there, and it is difficult for them to travel and they do not even know what a planned home birth is, because no one talks about it. No one even promotes it during prenatal care, so I repeat that everything starts with Primary Care.* (OB10)

*I see it as precarious due to the lack of support. I think women don’t have access, it’s a distant reality in Brazil. The basic unit, which should encourage it, sometimes doesn’t talk about it, doesn’t publicize it.* (OB11)

Home birth is often idealized as a unique, intimate and humanized experience; however, in the statements, it is observed that the practical reality in Brazilian society is far from allowing the choice of PHB in a safe and accessible manner. There is a need for significant changes, such as the creation of public policies to support and train healthcare professionals, as shown in the following statements:

*In theory, it’s great, but in practice, it’s a bit complicated. Here in Brazil, unfortunately, people nowadays don’t have access to a natural birth unless they have money. So, it’s hard for us to talk about home birth when we don’t even have prenatal care, so we’re very far from that, very far indeed.* (OB6)

*I think it is possible, yes, but it involves finding a balance, allowing women to have the freedom to choose this type of birth, as long as they have access to the necessary information and adequate support. For this to happen, it will take time, and it depends on the government.* (OB12)

*I think the government should invest more in training professionals and create public policies so that all women can have this option safely.* (OB13)

Thus, the meanings attributed by participants show that the Brazilian reality of PHB needs to be transformed for greater accessibility, especially with public policies for childbirth and professional training.

### Risk and safety in planned home birth

To carry out PHB, there are major obstacles to be overcome, such as residences’ inadequate conditions, which are often small and lack appropriate environments for the procedure, as per the following statements:

*In a planned home birth, there is a lot of preparation that is sometimes not possible. You see a house that has a living room, a bedroom, three rooms, let’s say, how are you going to prepare a room like that for the birth?* (OB2)

*Ah, home birth, you see something like this: the person lives there in a place that is no longer a good place to live, right? They don’t have access to several basic things: sanitation, water. There is no way to have the child at home.* (OB5)

*Spaces, I believe it is the space that is lacking.* (OB10)

There is significant cultural and professional resistance, expressed through fears and prejudices, which consequently creates difficulty in accepting PHB, according to the statements:

*I think that’s it, the knowledge and professionals too, I think they should be prepared and also want to, because I think I also see this as more difficult. I don’t see many professionals adhering to this issue of home birth today here in Brazil.* (OB4)

*And look, there is resistance from some healthcare professionals, who see risks and complications in this story. Some do not accept it due to their own experiences, others, due to comments.* (OB13)

The presence of a prepared multidisciplinary team is crucial to guarantee safe childbirth, but the training and availability of these professionals are still insufficient, and the availability of these professionals to work in a PHB is insufficient, as per the following statements:

*I think it’s precisely the lack of these teams, the lack of people who are qualified to provide this type of service. If a patient of mine who I do prenatal care for asks me, I have few people to recommend two or three who perform home births.* (OB6)

*If I can make this happen in a network, let’s say, combining a birthing center, a maternity ward, you know, I think I could ensure that it would be something safer.* (OB7)

*Pregnant women sometimes do not receive good prenatal care because there is a lack of nurses and physicians in basic units.* (OB10)

Thus, physicians express that the Brazilian context has many obstacles that make PHB unfeasible, especially in relation to the process safety, due to inappropriate and inadequate locations for the occurrence, fears and prejudices, in addition to the lack of a multidisciplinary team prepared to act in PHB assistance.

## DISCUSSION

Home birth is a type of birth that should be respected, considering women at low risk. In a study conducted by researchers in the Netherlands, which included a total of 80% of participants who were accompanied by healthcare professionals from the beginning of labor, the data on home births collected by the research team were compared with births that occurred in hospitals. Hence, the results of this research revealed that both types of birth had similar mortality rates, while the rates of invasive interventions were lower in PHB. Furthermore, no significant differences were observed in severe neonatal morbidity and mortality between the two groups studied^(18)^. The research concluded that, for women with low-risk births, PHB is a safe option, associated with minimal invasive medical interventions and perinatal morbidity and mortality rates no higher than those of births performed in hospitals.

The physicians interviewed warn about the risks inherent in PHB, significantly highlighting the need for hospital support to deal with serious complications. However, home birth, with the appropriate support and infrastructure, does not present a high risk compared to hospital births^(19)^. Another study, carried out in the Netherlands and using data from low-risk births across the country, proved that PHB does not present high risks, compared to hospital births, if there is all the appropriate support for the birth^(2)^. In this way, the meanings bring the negative risks of home birth, especially the risk regarding the place of birth. This brings to light the maintenance of a discourse of a hegemonic obstetric model, with the place of birth having to occur in a hospital environment, with the predominance of medical action.

But it is noted that the issue of risk mentions similarities with hospital birth, and in this sense, the maternal transfer rate during labor constitutes between 7.4% and 20%, which can contribute to a cesarean section that varies between 5.7% and 9%^(20)^. Thus, there is a similarity in the transfer rate of national and international studies, showing, in particular, that women who underwent cesarean section are within WHO recommendations regarding a rate of up to 15% of births. Thus, home birth and hospital birth do not have negative effects on issues of mortality, interventions, transfer or cesarean section, as they constitute similarities in these health indicators for obstetric care. However, this discourse in risk statements shows especially the established model and, especially, the absence of a physician in birth constitution.

Physicians report that for a safe PHB to occur, it is essential to provide adequate support, including appropriate infrastructure for women in labor. In this sense, a global systematic review compared the safety of PHB and hospital births, concluding that, although home birth can be safe, there are risks that can complicate the situation, as with any procedure. Thus, the research argues that the presence of adequate hospital infrastructure for emergency transfers, when necessary, is essential^(20)^. Furthermore, in another scientific study, which investigated the association between home births and the prevalence of neonatal hypoxic ischemic encephalopathy, the authors^(21)^ reported that the lack of infrastructure, especially the lack of a nearby safekeeping hospital to ensure maternal and neonatal safety, can impact the ability to respond quickly to obstetric emergencies^(21)^.

It is important to mention that there are countries that have adequate infrastructure to perform PHB, while others have significant gaps. In this regard, a study analyzed Brazilian demographic characteristics and perinatal outcomes of planned births in Brazil. The authors^(20)^ highlighted the challenges faced by the scarcity of resources available for performing home births. Thus, the research emphasized some needs, highlighting mainly the lack of an integrated network that can include nearby hospitals for emergencies. Thus, for PHB to be conducted effectively and safely, it is crucial that infrastructure is appropriate and that there are specialized and trained teams for this type of birth. This approach was used in a study in the United States that compared births in two environments, hospital and home, highlighting the importance of all professionals involved in home birth being professionally trained^(20)^.

It is crucial to emphasize that all healthcare professionals play an essential role in conducting PHB. A study analyzed the current overview of obstetric care for carrying out PHB and found a lack of trained teams and the need for specific training, representing significant challenges to be overcome^(22)^. Mention is made of the prohibition of the Federal Council of Medicine of Rio de Janeiro, with Resolution 348/2023^(23)^, which vetoed the participation of physicians in direct or indirect assistance in PHB, currently suspended by force of a judicial measure. Thus, there is an increasing attempt to inhibit healthcare professionals to guarantee women’s autonomy of choice in PHB.

Therefore, it is important to emphasize COFEN Resolution 737 of February 2, 2024, which regulates nurses-midwives’ and midwives’ work in assisting women, newborns and families in PHB^(24)^. This movement with the Federal Nursing Council shows an important advance in guaranteeing the performance of obstetric nursing in PHB with the nullity of Resolution 348/2023.

It is important to note that not all women choose to have a home birth, but rather women with high purchasing power, a specific group that holds this power of choice, due to the absence of measures that guarantee equity in home birth within the Brazilian Health System (In Portuguese, *Sistema Único de Saúde* - SUS)^(4)^. The demand is due to the humanized and respectful way in which women are treated during childbirth, in addition to having qualified and trained professionals to carry out any obstetric emergency, making PHB as safe as possible and with positive results for obstetric care^(1-4)^. Thus, PHB is not organized in the SUS healthcare network and is not part of health procedures. This statement significantly restricts access for Brazilian women. Hence, women with greater purchasing power have better opportunities and can guarantee their desires.

In this sense, bearing in mind the evident economic constraints of the place of birth through home birth, considering the high costs involved, it is necessary to reflect on ways to facilitate greater access to all Brazilian women. The SUS does not offer, or even recommend, home birth; on the contrary, the official technical standards on the subject even question the safety of this type of birth^(14,25)^. The MoH informs that PHB is not part of public health policies and there is also no recommendation for nulliparous women at low risk who plan PHB, especially since evidence comes from studies in other countries and is not necessarily applicable to the health context in Brazil.

Given this fact, within the standards of obstetric safety, when pregnancy is of low risk without complications, it should clearly be up to women to choose the place of birth. However, the current technocentric model, which prioritizes hospital environments and medical activities, does not allow progress towards guaranteeing women’s access and rights, maintaining a hegemonic and normative standard of care in obstetrics.

In the context of prenatal care, it is important to inform women about home birth, as occurs in other countries, such as the Netherlands and the United Kingdom, where primary care constitutes a link for carrying out a home birth for women at low risk within the health system^(6,7)^. Therefore, Brazil needs this articulation to raise awareness about home births, which currently face significant obstacles to ensuring women’s wishes are fulfilled.

This can be justified in two main ways: firstly, the SUS does not offer incentives or teams for home births^(25)^; secondly, this fact ends up excluding a large part of Brazilian women, due to the absence of public policies that guarantee home birth within the SUS^(4)^.

Physicians highlight the inadequate conditions for performing home births in poor homes without basic sanitation. According to those interviewed, these conditions make it impossible to perform the procedure. In Brazilian society, poor housing conditions are one of the obstacles to performing home births. Many homes do not have the necessary infrastructure to ensure safety during labor. This leads many women to prefer hospital births. In addition, home births are a limited and accessible option, especially for women who can afford care outside the SUS.

Although there are challenges associated with performing a home birth, there is also a strong social bias against this practice. Many parents face criticism from family and friends who consider home birth to be risky, preferring hospital birth, which is seen as safer. As a result, women who choose to give birth at home face social pressure against this choice^(20)^. Furthermore, there are medical professionals who are against this practice, demonstrating prejudice linked to PHB, which can negatively impact the decision of women who prefer PHB.

In fact, there are several barriers that impede the effectiveness of PHB. The lack of qualified teams is one of the main obstacles. The lack of public policies and adequate investment by government agencies in the training of these professionals result in poor professional training. Training generally occurs through courses offered during undergraduate studies and through continuing education in specializations, making it difficult to expand and ensure the safety of PHB. This contributes to the lack of professionals qualified to effectively carry out PHB.

### Study limitations

The study had as a limitation the lack of generalizability of results, inherent in the fact that the interviews were conducted with obstetricians from a hospital unit in northern Brazil, constituting a local reality that presents different cultural issues and specificities regarding the topic. Furthermore, there was difficulty regarding the time of conducting the interviews, since all the interviews occurred during the work period.

### Contributions to nursing, health or public policy

Knowledge of the meanings attributed by obstetricians to home birth is essential to promote discussions in favor of women’s autonomy and also for other non-medical professionals, such as nurses-midwives, to guarantee their rights in their professional practice. In many cases, there are perspectives in the medical profession that are contrary to their autonomy and established rights, but there must be changes in the hegemonic obstetric model in Brazil.

In this way, the study strengthens women’s right to natural childbirth and the guarantee of obstetric nursing in their professional practice, given the position against this practice, especially by medicine and its professional council.

## FINAL CONSIDERATIONS

It is understood that, based on the meanings attributed by obstetricians, home birth constitutes a risk factor, especially due to the lack of structure and support in the Brazilian health system. Thus, there is a need to rethink, due to scientific findings on the subject, and provide comprehensive support, as a health policy, for its implementation, in accordance with equity for home birth.

PHB can be a safe option for women with low-risk pregnancies, if adequate infrastructure and professional support are provided. However, the safety of PHB depends crucially on the availability of emergency support and an integrated health network that allows rapid transfers to hospitals when necessary.

In the Brazilian context, the viability of PHB is limited by significant challenges, including the lack of infrastructure, the absence of supportive public policies, and economic constraints, which exclude most women who depend on the SUS. Moreover, there is strong social and institutional prejudice against PHB, discouraging the choice of this mode of delivery.

To overcome these barriers, it is therefore necessary to invest in the training and qualification of healthcare professionals, develop public policies that integrate PHB as a safe and viable option, and raise awareness about the benefits and risks of this practice. Ensuring that women have access to information from the beginning of pregnancy is essential to being able to make informed choices about the place and mode of delivery. With a more inclusive and prepared health system, PHB can become a safe and desirable alternative for many women, respecting their preferences and needs.

## Data Availability

The research data are available only upon request.
