# Supplementary material for: The meanings attributed by obstetricians to planned home birth
Source: Rev Bras Enferm. 2025 Nov 7;78(4):e20240434. doi: 10.1590/0034-7167-2024-0434 (PMC12594481; doi:10.1590/0034-7167-2024-0434)
Supplement: Translation

# Translation: Os significados atribuídos por médicos obstetras sobre o parto domiciliar planejado

**DOI:** 10.1590/0034-7167-2024-0434pt

**Authors:** Laiane Ribeiro Viana, Diego Pereira Rodrigues, Valdecyr Herdy Alves, Bianca Dargam Gomes Vieira, Ediane de Andrade Ferreira, Giovanna Rosario Soanno Marchiori, Márcia Vieira dos Santos, Luana Asturiano da Silva

**Affiliations:** I Universidade Federal do Pará. Belém, Pará, Brasil; II Universidade Federal Fluminense. Niterói, Rio de Janeiro, Brasil; III Universidade Federal do Amapá. Macapá, Amapá, Brasil; IV Universidade Federal de Roraima. Boa Vista, Roraima, Brasil; V Hospital Servidores do Estado. Rio de Janeiro, Rio de Janeiro, Brasil

**Keywords:** Parto Domiciliar, Médicos, Obstetra, Parto Humanizado, Política de Saúde.

## Abstract

**Objetivos:**

compreender os significados atribuídos por médicos obstetras sobre o parto domiciliar planejado.

**Métodos:**

trata-se de estudo qualitativo, realizado com 13 médicos obstetras de um hospital regional de Belém, Pará, recrutados pela técnica *snowball sampling*. Foi utilizada entrevista semiestruturada, gravada e transcrita na íntegra para a realização da análise de conteúdo com apoio do *software* ATLAS ti 22.7.

**Resultados:**

observou-se falta de dimensionamento, maternidades de suporte e poder aquisitivo elevado para garantir o parto. Além da falta de estrutura e de profissionais, a ausência de conhecimento sobre o tema e de políticas públicas constitui obstáculo no âmbito da saúde brasileira.

**Considerações Finais:**

compreende-se que, pelos significados atribuídos pelos médicos obstetras, o parto domiciliar constitui um fator de risco, especialmente pela falta de estrutura e suporte no sistema de saúde brasileiro.

## INTRODUÇÃO

Até o início do século XIX, havia uma predominância de assistência ao parto por parteiras. Mas essa realidade mudou drasticamente com a criação dos hospitais, promovendo uma bandeira de maior segurança e redução de mortalidade perinatal^(1)^. Logo, o processo de parto sofreu mudanças drásticas, as quais, por vezes, podem ser vistas como uma redução da autonomia da mulher sobre as escolhas quanto à condução de todo esse momento, o qual deveria ter um foco nas decisões da parturiente.

Esse panorama obstétrico exerce intensas intervenções sobre o corpo feminino, como medicalização, episiotomia, manobra de Kristeller, cesariana e outras práticas introduzidas, e às vezes, são utilizadas sem nenhuma justificativa clínica verificável^(2,3)^, comprometendo, por vezes, todo um planejamento pessoal da gestante quanto a realização do parto.

Com esse efeito, em meados do século XXI, com a popularização da internet e a propagação de informações, houve o compartilhamento de experiências e trocas do movimento de mulheres, buscando alternativas contra os abusos cometidos nas instituições de saúde. O parto domiciliar planejado (PDP) surge para romper com o modelo biomédico e intervencionista, garantindo melhores cuidados e respeito às mulheres.

O âmbito do nascimento no Brasil é retratado com uma predominância de parto hospitalar, com menos de 2,4% de parto domiciliares^(4)^. No estado do Pará, também tem essa semelhança, mas com diferença na realidade, pois os partos domiciliares na região amazônica são culturais, principalmente em locais com infraestrutura limitada, que muitas vezes contam com uma parteira tradicional, seja em comunidades com populações tradicionais, como ribeirinhas, quilombolas ou indígenas. Culturalmente, há uma carência de centros urbanos e unidades assistenciais, e, assim, detém, a partir da prática das parteiras tradicionais, os cuidados das mulheres no âmbito de nascimento^(5)^. Outro fato também é que o estado não detém os dados de equipes especializadas no parto domiciliar, como médicos e enfermeiros, em que há inciativas de poucas equipes particulares, que não são todas as mulheres que têm as condições financeiras de contratação.

Assim, podemos destacar que, em vários países, as mulheres dão à luz por meio do PDP, como Reino Unido, Canadá, Austrália, Holanda, Suécia, Nova Zelândia, entre outros. A assistência se mostra tão segura como em nascimentos em unidades hospitalares^(6)^. Dois estudos destacam a segurança dos partos domiciliares de risco habitual assistidos por obstetrizes/enfermeiras obstétricas em áreas onde essa modalidade está integrada ao sistema de saúde^(7)^. Assim, não se encontraram diferenças na mortalidade perinatal/neonatal em seu estudo de coorte de 11.493 nascimentos vs. 11.493 nascimentos hospitalares em Ontário, Canadá. Meta-análise concluiu que as evidências sobre os resultados maternos apoiam consistentemente os PDP nessas condições^(8)^.

Desse modo, há uma demanda cada vez maior por um modelo que rompa com os serviços tradicionais, com normas institucionais rígidas, e que promova uma assistência mais individualizada, com escuta qualificada e confiança nas reais necessidades das mulheres e suas famílias, tornando a escolha do local de parto uma decisão informada, consciente e intuitiva^(9)^. Neste sentido, a Organização Mundial da Saúde (OMS), o *American College of Obstetricians and Gynecologists* (ACOG) e o *National Institute for Health and Care Excellence* (NICE) reafirmam a segurança do PDP, com menos intervenções e menos associações de lacerações, infecções e morbidade^(8)^. Dessa forma, incentivam e recomendam às mulheres de risco habitual dar à luz por essa experiência, garantindo maior respeito e autonomia^(10-13)^.

Mas no cenário brasileiro, o Ministério da Saúde (MS), por meio da Nota Técnica nº 2/2021 do Departamento de Ações Programáticas Estratégicas da Secretaria de Atenção Primária à Saúde, fragiliza a autonomia das mulheres quanto à escolha do parto domiciliar, bem como a atuação de profissionais de saúde, como a enfermeira obstétrica. A referida nota técnica reforça o modelo obstétrico hegemônico centrado no saber-médico, hospitalocêntrico e tecnocrático, que afirma não haver grandes estudos clínicos randomizados, com metodologia rigorosa, que corroborem a segurança do nascimento em ambiente domiciliar, desaconselhando, assim, a escolha do parto domiciliar como local de nascimento^(14)^.

Mostra-se necessário reafirmar que esta nota técnica está alinhada a outras medidas do governo federal (2019-2022), como a nova Caderneta da Gestante, as boas práticas baseadas em evidências científicas, a Rede de Atenção à Saúde Materno-Infantil, que são contrárias aos direitos das mulheres, além de profissionais não médicos. Esse fato se baseia em um posicionamento cooperativista da medicina que repercute diretamente no poder de escolha das mulheres do governo brasileiro. Com o novo governo progressista, revogaram-se todas as medidas que criavam obstáculos para a saúde reprodutiva e também para atuação de outros profissionais de saúde. Atualmente, o governo brasileiro instituiu a Rede Alyne em prol dos Objetivos de Desenvolvimento Sustentável da Agenda 2030 com a mortalidade materna e rompendo todos os retrocessos de políticas e iniciativas para a saúde das mulheres.

Dessa forma, a nota técnica do governo brasileiro em 2021 propaga o modelo hegemônico da assistência obstétrica e não garante o PDP, mesmo que instituições de reconhecimento, como a OMS, ACOG e NICE, recomendaram tal realização. Um fato ainda é que a Agência Nacional de Saúde Suplementar não garantir, no rol de procedimentos, o PDP mostra a total desconexão do MS com as recomendações internacionais e as evidências científicas, com apenas a manutenção do modelo assistencial.

Mesmo assim, faz-se necessário compreender os significados dos médicos obstetras quanto à sua percepção do PDP, visto que pode ocorrer a entrada de mulheres na rede obstétrica por complicações decorrentes da assistência, e os seus significados demonstram a compreensão do cerne deste fenômeno, mesmo com essa assistência não estando interligada diretamente à rede de atenção obstétrica. Nesse sentido, o estudo tem como questão norteadora: quais os significados atribuídos por médicos obstetras atuantes em unidades hospitalares sobre o PDP?

## OBJETIVOS

Compreender os significados atribuídos por médicos obstetras sobre o PDP.

## MÉTODOS

### Aspectos éticos

O estudo foi aprovado pelo Comitê de Ética em Pesquisa do Instituto de Ciências da Saúde da Universidade Federal do Pará, conforme disposto na Resolução nº 466/2012 do Conselho Nacional de Saúde. Para preservar o sigilo, o anonimato e a confiabilidade, as depoentes foram identificadas pelas letras “MO” de médico obstetra, seguidas de algarismo numérico, correspondente à sequência da realização das entrevistas (MO1, MO2, MO3, …, MO13), além da garantia da participação voluntária, mediante assinatura do Termo de Consentimento Livre e Esclarecido.

### Tipo de estudo

Trata-se de estudo de abordagem qualitativa, de caráter descritivo, exploratório. A forma de recrutamento dos participantes foi realizada pela técnica *snowball sampling*, conhecida também como bola de neve^(15)^. Essa técnica utilizada é realizada com a indicação de sementes por parte de cada entrevistado, com a indicação por parte dele, com a produção de novos contatos do processo de recrutamento dos participantes^(15)^. Para realização do rigor metodológico, foi utilizado o instrumento *COnsolidated criteria for REporting Qualitative research* (COREQ), que constituiu a ferramenta para o desenvolvimento desta pesquisa qualitativa.

### Participantes e fontes de dados

O estudo foi realizado com 13 médicos obstetras atuantes no Hospital Regional Abelardo Santos, situado no município de Belém, Pará, Brasil. A unidade de saúde constitui um serviço público do estado do Pará, sendo um dos principais hospitais na Região Metropolitana para a assistência obstétrica de gestação de risco habitual.

Foi iniciado o processo de ambientação, com a apresentação do estudo à coordenação da unidade. Com isso, foram solicitados à coordenação três contatos dos médicos. Em posse deste contato, foi iniciada a interação inicial com eles, por meio do aplicativo *WhatsApp^®^*, sendo esclarecidos os temas, objetivos, técnicas de coleta de dados, riscos e benefícios do estudo. Após esse processo, os critérios de inclusão foram ser médico obstetra e estar atuando no centro obstétrico. Os critérios de exclusão foram ser profissionais de saúde de outras áreas da saúde. Ressalta-se que nenhum participante foi excluído do estudo.

Após essa aplicação, foram agendadas na unidade as entrevistas com cada participante. Após a realização da entrevista, o participante fornecia mais três contatos para realizar o processo de recrutamento, conforme a técnica *snowball sampling*, até o estabelecimento do número de participantes do estudo.

O encerramento da coleta de dados e o estabelecimento do número de 13 participantes do estudo foram feitos por meio do processo de saturação teórica^(16)^, que se refere quando os dados não trazem elementos novos para balizar ou aprofundar a teorização^(16)^, dando, assim, um cerne de significado sobre o problema. Desse modo, quando se chegou ao número de dez participantes, sucedeu que os dados não estavam dando nenhum elemento novo, e, assim, estabeleceram-se mais três participantes, sucedendo o número determinado de participantes do estudo.

### Coleta e organização de dados

Foi utilizada como técnica de coleta de dados a entrevista semiestruturada, com perguntas abertas e fechadas, realizada no período de novembro de 2023 a março de 2024, que durou em média 45 minutos. As entrevistas ocorreram individualmente, em um momento único, com sala reservada na unidade hospitalar, contendo apenas a participação da pesquisadora principal e do entrevistado, para realização por apenas um entrevistador durante a aplicação da técnica de coleta de dados.

A entrevista semiestruturada continha informações sobre os dados socioeconômicos, educacionais e profissionais, e duas perguntas disparadoras, a saber: qual a sua percepção sobre o PDP no contexto brasileiro? Quais os principais obstáculos no acesso e assistência ao PDP? Cada entrevista teve a utilização do recurso de gravação por MP3 para a captação do depoimento, com a devida autorização prévia dos participantes.

Os depoimentos foram transcritos na íntegra. Depois dessa etapa, foram validados pelos entrevistados, mostrando em outro momento o envio de seus discursos transcritos pelo aplicativo *WhatsApp* ^®^, ocorrendo, assim, a sua validação dos dados, como determina o COREQ.

Ressalta-se que ocorreu a realização de estudo piloto com três participantes, que não foram contabilizados no estudo, que teve a finalidade de verificar a adequação da entrevista com os objetivos da pesquisa e dos depoimentos dos médicos.

A coleta de dados foi conduzida somente por um pesquisador, apresentando domínio da técnica empregada, evitando, assim, uma condução diferente, enquanto a equipe de investigação ficará na competência de atuação no tratamento e análise dos dados.

### Análise dos dados

Foi realizada a análise de conteúdo^(17)^ na organização e tratamento dos dados. Primeiro, ocorreu a realização da primeira etapa, a pré-análise, com a leitura flutuante e estabelecimento do contato inicial com os dados, buscando a primeira mensagem obtida, com a escolha de documentos que irão compor o *corpus* do estudo, de acordo com dados na exaustividade, representatividade, homogeneidade e pertinência^(17)^.

Em seguida, para a exploração do material^(17)^, foram construídas as intervenções de codificação. Assim, a funcionalidade do *software* ATLAS.ti 22.7 foi submetida com a análise indutiva, diante da codificação dos segmentos dos depoimentos com a assimilação dos códigos e concepção das composições, a saber: risco obstétrico; insuficiência de estrutura; falta de profissionais capacitados; elevado poder aquisitivo; falta de informação; políticas públicas; e deficiência de equipes profissionais, com o propósito da busca da unidade de significação a partir dos códigos que surgiram do processo.

No último polo cronológico do processo analítico, tratamento dos resultados, inferência e interpretação, classificaram-se os elementos construtivos pelo processo de exclusão mútua, como homogeneidade, pertinência, objetividade, fidelidade e produtividade, visando à categorização^(17)^. Nessa última etapa do procedimento indutivo de análise, foram submetidos os conteúdos coletados, sendo cada um vinculado aos documentos do termo, com a sigla acompanhada de numeração, conforme e adequada ao *software*, iniciando de D1 a D15. A seguir, cunharam-se as citações de componentes dos documentos, de tal modo que estas se elencaram com códigos, que constituíram nomeados de composição com o sentido da citação decodificada pelo pesquisador.

A partir dessa etapa, ocorreram a contagem de códigos imperativos, a combinação com as temáticas indutivas emergentes de forma majoritária nas entrevistas e, posteriormente, a saturação desses códigos, por meio da recorrência de significados, exprimindo a concretização de um dicionário de códigos. Estes permitem a concepção do conjunto de códigos e as citações correspondentes, através da assimilação, dessa forma, das unidades de significados, com a classificação das informações construtivas e o reagrupamento dos significados, que surgem da conjunção dos retornos dos participantes, motivando a construção das categorias^(17)^.

Com esse processo, foram construídas as seguintes categorias: 1) Os significados do parto domiciliar planejado no Brasil; 2) Desafios e realidades do acesso ao parto domiciliar planejado no contexto brasileiro; 3) Risco e segurança no parto domiciliar planejado. A discussão teve como base as políticas públicas no campo do parto e nascimento e a literatura científica no tema do PDP.

## RESULTADOS

As 13 entrevistas realizadas revelaram diversas características demográficas, educacionais e profissionais dos participantes. A distribuição por sexo mostra que dez são do gênero feminino, e três, do masculino. Em relação à idade, sete possuem 20 e 30 anos, quatro, entre 40 e 50 anos, e dois, mais de 50 anos.

Quanto à raça/etnia, seis se autodeclaram como brancos, seis, como pardos, e um, como preto. As afiliações religiosas são variadas, com cinco católicos, dois evangélicos, dois sem religião, dois cristãos (sem especificar denominação), um espírita e um sem religião especificada. Quanto ao estado civil, seis são solteiros, seis, casados, e um, divorciado.

Todos possuem formação em medicina, sendo oito em instituições públicas, e cinco, em particulares. As instituições de formação incluem o Centro Universitário do Pará, a Universidade Federal do Pará, o Centro Universitário Unifamaz e a Universidade Estadual do Pará.

No campo das especializações, todos tinham especializações. Nenhum dos entrevistados possuía curso de pós-graduação *stricto sensu*.

### Os significados do parto domiciliar planejado no Brasil

Segundo os significados atribuídos pelos médicos obstetras, mencionam o risco inerente ao PDP, como hemorragia, distocia e complicações imprevistas. Desse modo, mostra-se que, a partir de seus significados, um aspecto negativo por parte dos médicos, especialmente com a escassez de suporte imediato em casos de emergência, é preocupante, conforme os depoimentos:

*Olha, acho perigoso, porque o ideal é a paciente estar no hospital caso tenha uma hemorragia puerperal, que é uma das principais complicações do parto normal, então acho perigoso, porque aí ela não vai ter uma assistência naquele momento que ela precisa, caso tenha uma complicação pós-parto.* (MO1)

*Olha, quero lhe dizer, não tenho uma boa concepção, aceitação, vamos dizer, do parto domiciliar em virtude dos riscos, entendeu? Porque é uma incógnita. Naquela hora, pode dar tudo certo, como podem sair coisas erradas, você coloca em risco a vida de uma mulher e de uma criança* [...] *de repente, o parto vai tudo certo, e aí a placenta fica presa e dá hemorragia, e coloca ela em risco e você não pode! É questão de pouco tempo que você tem para ir da sua casa para um hospital.* (MO2)

*Mas ele também pode complicar e precisar de uma assistência hospitalar e tudo mais, acho arriscado, no Brasil, no contexto do Brasil, visando, assim, nosso sistema de saúde. Geralmente, nossa formação, eu acho arriscado. Tem mais risco de complicação para o nascimento do bebê.* (MO4)

O PDP encontra barreiras, conforme os depoentes, como a insuficiência de infraestrutura adequada para os partos domiciliares no contexto brasileiro, especialmente pela falta de unidades hospitalares, equipamentos escassos e dimensionamento de pessoal, profissionais treinados nas emergências das maternidades, segundo os depoimentos a seguir:

*Teria que ter um hospital de retaguarda. Se tivesse um hospital de retaguarda próximo aos locais a domicílio, isso poderia contribuir de uma forma positiva, não é? Caso ela tenha um parto em casa, caso tenha alguma complicação, ela ir para o hospital próximo para ter ajuda.* (MO1)

*Não tem o suporte médico normalmente, entendeu?* [...] *se não tiver perto de uma unidade hospitalar e pronto atendimento de urgência e emergência obstétrica, acho perigoso... a maioria das casas de parto ainda não tem um profissional médico de sobreaviso, o que é triste.* (MO3)

*Uma hemorragia puerperal que você tem que agir imediatamente, não é? É difícil você atuar num domicílio se você não tem uma ambulância ali para levar ela rapidamente para o hospital.* (MO6)

*Parto domiciliar é uma realidade distante nos princípios do SUS, porque tudo começa na unidade básica, e o atendimento é precário e não tem assistência.* (MO10)

No dimensionamento pessoal, há uma necessidade de profissionais com experiência e preparados para conduzir partos domiciliares. Assim, os depoentes mencionam frequentemente a comparação com outros países, em que há uma preocupação e apoio aos profissionais, conforme os depoimentos a seguir:

*Perigoso, não tem o suporte médico normalmente, entendeu?* [...] *até a Inglaterra, que tem as enfermeiras obstétricas treinadas para parto normal, elas sempre têm um médico de sobreaviso, sempre, não é a nossa realidade aqui no brasil ainda, infelizmente, ainda não, entendeu?* [...] *a maioria das casas de parto ainda não tem um profissional médico de sobreaviso, o que é triste, entendeu?* [...] *não que a enfermagem obstetra não seja capaz de desfazer, por exemplo, uma intercorrência durante o parto vaginal, mas acho que, no ambiente domiciliar, é um risco desnecessário.* (MO3)

*É preciso, na verdade, que os profissionais que estejam encarregados sejam bastantes experientes. Acho que é preciso uma equipe, porque o parto domiciliar pode dar muito certo, não é se* […] *mas ele também pode complicar e precisar de uma assistência hospitalar e tudo mais* [...] *no Brasil, contexto Brasil, visando, assim, nosso sistema de saúde, geralmente nossa formação acho arriscado.* (MO4)

*A pessoa que faça a doula, no caso o parto domiciliar, ela tenha, além da experiência, o conhecimento em saber até onde ela pode ir.* (MO8)

Os significados atribuídos pelos depoentes mostram ser o PDP mais viável para as mulheres com poder aquisitivo elevado, o que não reflete a realidade da população atendida pelo SUS, como mostram os depoimentos a seguir:

*Quando penso em trabalho de parto planejado para acontecer em meio domiciliar, estou pensando em gente que tem dinheiro para poder ter tudo; não estou pensando na pessoa que é assistida pelo SUS.* (MO7)

*Muita gente não sabe, só tem uma base do que é no pré-natal, não é falado ainda. São precários os investimentos também, até a casa da pessoa às vezes é precária, você sabe a realidade do nosso país, então elas acabam vindo para o hospital onde se sentem seguras caso tenha intercorrências.* (MO11)

Desse modo, os entrevistados mostram, com base em sua percepção, os significados negativos quanto ao PDP no âmbito da assistência obstétrica no contexto brasileiro.

### Desafios e realidades do acesso ao parto domiciliar planejado no contexto brasileiro

É importante mencionar que os depoimentos fazem uma comparação entre as áreas rural e urbana, ou seja, nas áreas afastadas das cidades, existe maior acesso ao parto domiciliar por essa particularidade de falta de recursos e estrutura, o que pode promover riscos elevados para a mãe e o bebê. Outrossim, nas grandes cidades, a facilidade do acesso aos hospitais torna a escolha pelo parto hospitalar a escolha predominante:

*Eu acho que é mais facilitado em regiões que não tem hospital, tipo interior, que tem muitas parteiras. Acho que, nesses locais, deve ser mais fácil o acesso. Na cidade grande, acho que é mais fácil ir para o hospital.* (MO1)

*O acesso, acho que não é muito incentivado, não vejo assim pelo menos aqui na nossa região, não é? O parto domiciliar não é incentivado. Mesmo as pessoas trabalhando com isso, até existem grupos de parto normal, mas sempre são realizados dentro do hospital.* (MO4)

*Precário porque não tem suporte, nem pré-natal é feito direito. Então, eu vejo que não tem acesso. Vemos mais para o interior, mas não tem acesso é o que tem lá e fica difícil o deslocamento para elas, e elas nem sabem o que é parto domiciliar planejado, porque ninguém fala, ninguém divulga, nem no pré-natal, então eu volto a dizer que tudo começa na atenção básica.* (MO10)

A falta de conhecimento e de divulgação adequada pelas Unidades Básicas de Saúde atrelada ao PDP e à existência de tabus em torno desse tema se configura como barreira. Isso significa que muitas mulheres não são informadas sobre a possibilidade de o parto ser domiciliar planejado, conforme os depoimentos:

*Ainda existe um certo tabu. Acho que as próprias gestantes não sabem sobre o parto domiciliar.* (MO5)

*Precário porque não tem suporte, mas não é acesso é o que tem lá, e fica difícil o deslocamento para elas e elas nem sabem o que é parto domiciliar planejado, porque ninguém fala. Ninguém divulga nem no pré-natal, então eu volto a dizer que tudo começa na Atenção Básica.* (MO10)

*Eu vejo como precário por falta de suporte. Acho que as mulheres não têm acesso, é uma realidade distante no Brasil. A unidade básica, que deveria incentivar às vezes, não fala, não divulga.* (MO11)

O parto domiciliar é frequentemente idealizado como uma experiência única, intimista e humanizada, entretanto, nos depoimentos, observa-se que a realidade prática na sociedade brasileira está distante de permitir a escolha pelo PDP de forma segura e acessível. Torna-se preciso haver mudanças significativas, como a criação de políticas públicas de apoio e capacitação dos profissionais de saúde, conforme mostram os depoimentos a seguir:

*Na teoria, é muito bom, mas, na prática, é um pouco complicado. Aqui no Brasil, infelizmente, o parto normal, hoje em dia, a pessoa, assim, não tem um acesso a um tipo de parto humanizado se ela não tiver dinheiro. Então, é difícil a gente falar em parto domiciliar quando a gente não tem nem assistência no pré-natal, então a gente está muito distante disso, muito distante mesmo.* (MO6)

*Acho que é possível, sim, mas envolve a busca de um equilíbrio, permitindo que as mulheres tenham a liberdade de escolher esse tipo de parto, desde que tenham acesso à informação necessária e ao suporte adequado. Para isso acontecer, vai demorar, depende da esfera governamental.* (MO12)

*Acho que o governo deveria investir mais em capacitação de profissionais e criar políticas públicas para que todas as mulheres possam ter essa opção de maneira segura.* (MO13)

Assim, os significados atribuídos pelos participantes mostram que a realidade brasileira do PDP necessita ser transformada para maior acessibilidade, especialmente com políticas públicas para o parto e formação profissional.

### Risco e segurança no parto domiciliar planejado

Para efetuar o PDP, há grandes obstáculos a serem enfrentados, como as condições inadequadas das residências que, muitas das vezes, são pequenas e carecem de ambientes apropriados para o procedimento, conforme os depoimentos a seguir:

*No parto planejado domiciliar, tem todo um preparo que às vezes não é possível. Tu vês uma casa que tem uma sala, um quarto, três cômodos, vamos dizer, como é que tu vais preparar um cômodo desse para fazer o parto.* (MO2)

*Ah, parto domiciliar, tu vês uma coisa assim: a pessoa mora ali num local que já não é legal para morar, não é? Não tem acesso a várias coisas básicas: saneamento, água. Não tem como ter o filho em casa.* (MO5)

*Espaços, acredito que seja o espaço que não tem.* (MO10)

Há uma resistência cultural e profissional significativa, expressada por medos e preconceitos, que consequentemente engendra dificuldade de aceitação do PDP, conforme os depoimentos:

*Eu acho que é isso, o conhecimento e os profissionais também, acho que devem estar preparados e também quererem, porque acho que vejo também mais difícil isso. Não vejo muito os profissionais aderirem a essa questão do parto domiciliar hoje aqui no Brasil.* (MO4)

*E olha, tem uma resistência por parte de alguns profissionais de saúde, que enxergam riscos e complicações nessa história. Alguns não aceitam por experiências próprias, outros, por comentários.* (MO13)

A presença de uma equipe multidisciplinar preparada é crucial para garantir a segurança do parto, mas a formação e disponibilidade desses profissionais ainda são insuficientes, e a disponibilidade desses profissionais de atuarem em um PDP é insuficiente, conforme os depoimentos a seguir:

*Acho que justamente a falta dessas equipes, falta de pessoas mesmo capacitadas para fazer esse tipo de serviço. Se uma paciente minha que eu faço pré-natal, se uma paciente minha perguntar, tenho poucas pessoas para indicar duas ou três que fazem o parto domiciliar.* (MO6)

*Se eu conseguir fazer com que isso fosse feito em rede, vamos dizer assim, conciliar casa de parto, maternidade, sabe, acho que eu conseguiria garantir que fosse algo mais seguro.* (MO7)

*As gestantes não têm um bom pré-natal às vezes porque faltam enfermeiros e médicos nas unidades básicas.* (MO10)

Desse modo, os médicos expressam que o contexto brasileiro detém muitos obstáculos que inviabilizam o PDP, especialmente em relação à segurança do processo, por conta de locais impróprios e inadequados para a ocorrência, medos e preconceitos, além da falta de equipe multiprofissional preparada para atuar na assistência ao PDP.

## DISCUSSÃO

O parto domiciliar constitui uma modalidade de nascimento que deve ser respeitada, considerando as mulheres de risco habitual. Em estudo conduzido por pesquisadores na Holanda, que abrangeu um total de 80% das participantes acompanhadas por profissionais de saúde desde o início do trabalho de parto, os dados sobre partos domiciliares coletados pela equipe de pesquisa foram comparados com os partos ocorridos em hospitais. Nesse sentido, os resultados desta pesquisa revelaram que ambas as modalidades de parto apresentaram taxas de mortalidade semelhantes, enquanto as taxas de intervenções invasivas foram menores nos PDP. Além disso, não foram observadas diferenças significativas na morbimortalidade neonatal grave entre os dois grupos estudados^(18)^. A pesquisa concluiu que, para mulheres com parto de risco habitual, o PDP constitui uma opção segura, associada a intervenções médicas invasivas mínimas e taxas de morbimortalidade e mortalidade perinatal não superiores às dos partos realizados em hospitais.

Os médicos entrevistados alertam sobre os riscos inerentes ao PDP, destacando significativamente a necessidade de suporte hospitalar para lidar com complicações graves. No entanto, o parto domiciliar, com o devido suporte e infraestrutura, não apresenta risco elevado em comparação aos partos hospitalares^(19)^. Outra pesquisa, realizada na Holanda e utilizando dados de nascimentos de baixo risco em todo o país, comprovou que o PDP não apresenta riscos elevados, em comparação aos partos hospitalares, caso haja todo o suporte adequado para a realização do parto^(2)^. Dessa forma, os significados trazem os riscos negativos do parto domiciliar, especialmente ao risco quanto ao local de nascimento. Isso traz à tona a manutenção de um discurso do modelo hegemônico obstétrico, com o local de nascimento devendo ocorrer no ambiente hospitalar, com o predomínio da atuação médica.

Mas nota-se que a questão do risco menciona as semelhanças com o parto hospitalar, e neste sentido, a taxa de transferência materna durante o trabalho de parto constitui entre 7,4% e 20%, podendo contribuir para uma cesariana que varia entre 5,7% e 9%^(20)^. Assim, há uma similaridade de taxa de transferência de estudos nacionais e internacionais, mostrando, especialmente, que as mulheres que foram submetidas à cesariana estão dentro das recomendações da OMS quanto uma taxa de até 15% dos partos. Desse modo, o parto domiciliar e o hospitalar não têm efeitos negativos quanto às questões de mortalidade, intervenções, transferência ou cesariana, pois constituem semelhanças desses indicados de saúde para a assistência obstétrica. Mas esse discurso nos depoimentos de risco mostra especialmente o modelo instituído e, especialmente, a ausência do médico na constituição do nascimento.

Os médicos relatam que, para um PDP ocorrer de forma segura, é essencial oferecer suporte adequado, incluindo infraestrutura apropriada para as parturientes. Nesse sentido, revisão sistemática global comparou a segurança entre PDP e partos hospitalares, concluindo que, embora o parto domiciliar possa ser seguro, existem riscos que podem complicar a situação, assim como em qualquer procedimento. Desse modo, a pesquisa argumenta que é fundamental a presença de uma infraestrutura hospitalar adequada para transferências emergenciais, quando necessário^(20)^. Além disso, em outro estudo científico, que investigou a associação entre partos domiciliares e a prevalência de encefalopatia hipóxica isquêmica neonatal, os autores^(21)^ informaram que a ausência de infraestrutura, especialmente a falta de um hospital de resguardo próximo para garantir a segurança materna e neonatal, pode impactar a capacidade de resposta rápida a emergências obstétricas^(21)^.

É importante mencionar que existem países que possuem infraestruturas adequadas para realizar PDP, enquanto outros apresentam lacunas importantes. Nesse viés, estudo analisou as características demográficas brasileiras e os resultados perinatais de partos planejados no Brasil. Os autores^(20)^ evidenciaram os desafios enfrentados pela escassez de recursos disponíveis para a realização de partos domiciliares. Desse modo, a pesquisa enfatizou algumas necessidades, destacando principalmente a falta de uma rede integrada que possa incluir hospitais próximos para casos de emergências. Dessa forma, para que o PDP seja conduzido de forma eficaz e segura, é crucial que a infraestrutura seja apropriada e que haja a presença de equipes especializadas e capacitadas para essa modalidade de parto. Essa abordagem foi utilizada em estudo nos Estados Unidos que comparou partos nos dois ambientes, hospitalar e domiciliar, destacando a importância de todos os profissionais envolvidos no parto domiciliar serem capacitados profissionalmente^(20)^.

É crucial ressaltar que todos os profissionais de saúde desempenham um papel essencial na condução do PDP. Estudo analisou o panorama atual da assistência obstétrica para a realização do PDP e constatou a falta de equipes capacitadas e a necessidade de formação específica, representando desafios significativos a serem superados^(22)^. Menciona-se a proibição do Conselho Federal de Medicina do Rio de Janeiro, com a Resolução nº 348/2023^(23)^, que vetou a participação do médico na assistência direta ou indireta no PDP, atualmente suspensa por força de medida judicial. Nesse sentido, há, cada vez mais, uma tentativa de inibir profissionais de saúde para garantir a autonomia de escolha da mulher no PDP.

Dessa forma, é importante enfatizar a Resolução COFEN nº 737, de 02 de fevereiro de 2024, que normatiza a atuação do enfermeiro obstétrico e obstetriz na assistência à mulher, recém-nascido e família no PDP^(24)^. Esse movimento junto ao Conselho Federal de Enfermagem mostra um importante avanço para a garantia da atuação da enfermagem obstétrica no PDP com a nulidade da Resolução nº 348/2023.

Ressalta-se que não são todas as mulheres que têm essa escolha do parto domiciliar, mas sim mulheres com poder aquisitivo elevado, sendo um grupo específico que detém esse poder de escolha, pela ausência de medidas que garantam a equidade do parto domiciliar no âmbito do Sistema Único de Saúde (SUS)^(4)^. A procura se dá pela forma humanizada e respeitosa com a mulher durante o parto, além de ter profissionais capacitados e treinados para a execução de qualquer emergência obstétrica, fazendo com que o PDP seja tão seguro e com resultados positivos para a assistência obstétrica^(1-4)^. Assim, o PDP não está organizado na rede de atenção à saúde do SUS e não faz parte dos procedimentos de saúde. Essa afirmação restringe de forma considerável o acesso de mulheres brasileiras. Desse modo, as mulheres com maior poder aquisitivo detêm melhores oportunidades e garantem os seus desejos.

Nesse sentido, tendo em mente a restrição econômica evidente do local de nascimento por meio do parto domiciliar, considerando os altos custos envolvidos, é necessário refletir sobre maneiras de viabilizar um maior acesso a todas as mulheres brasileiras. O SUS não oferece, ou sequer indica, o parto domiciliar; pelo contrário, as normas técnicas oficiais sobre o assunto até questionam a segurança dessa via de parto^(14,25)^. O MS informa que o PDP não faz parte das políticas públicas de saúde e também não há recomendação das mulheres nulíparas de risco habitual que planejam o PDP, especialmente que as evidências são oriundas de estudos em outros países e não necessariamente aplicáveis ao contexto de saúde no Brasil.

Diante desse fato mencionado, dentro dos padrões de segurança obstétrico, quando a gestação for de risco habitual sem complicações, evidentemente deveria caber à mulher a escolha do local de nascimento. Mas o atual modelo tecnocêntrico vigente, o qual prioriza o ambiente hospitalar e as atividades médicas, não permite avançar para a garantia de seu acesso e direitos das mulheres, mantendo um padrão hegemônico e normativo de assistência na obstetrícia.

No âmbito do pré-natal, torna-se importante a informação de mulheres sobre o parto domiciliar, como ocorre em outros países, como Holanda e Reino Unido, que a atenção primária constitui um elo para realização de um parto domiciliar de mulheres de risco habitual dentro do sistema de saúde^(6,7)^. Dessa forma, o Brasil necessita dessa articulação para o conhecimento do parto domiciliar que, por ora, enfrenta obstáculos importantes para a garantia das mulheres quanto aos seus desejos.

Isso pode ser justificado de duas principais formas: primeiramente, o SUS não oferece incentivos ou equipes para realização de parto domiciliar^(25)^; segundamente, esse fato acaba excluindo grande parte das mulheres brasileiras, pela ausência de políticas públicas que garantam o parto domiciliar no âmbito do SUS^(4)^.

Os médicos destacam as condições inadequadas para realizar PDP em residências carentes, sem saneamento básico. Segundo os entrevistados, essas condições tornam inviável a realização do procedimento. Na sociedade brasileira, as condições precárias de moradia são um dos obstáculos para a realização do parto domiciliar. Muitas habitações não possuem a estrutura necessária para garantir a segurança durante o trabalho de parto. Isso leva muitas mulheres a preferirem o parto hospitalar. Além disso, o PDP é uma escolha limitada e acessível principalmente às mulheres que podem pagar por assistência fora do SUS.

Embora existam dificuldades relacionadas à realização do PDP, também há um forte preconceito social em relação a essa prática. Muitos pais enfrentam críticas de familiares e amigos que consideram o parto domiciliar arriscado, preferindo o parto hospitalar, visto como mais seguro. Como resultado, as mulheres que escolhem parir em casa enfrentam pressão social contra essa escolha^(20)^. Ademais, existem profissionais médicos que são contra essa prática, demonstrando preconceito ligado aos PDP, o que pode impactar negativamente a decisão das mulheres que preferem o PDP.

Efetivamente, há várias barreiras que impedem a eficácia do PDP. A falta de equipes qualificadas é um dos principais obstáculos. A escassez de políticas públicas e de investimentos adequados por parte das esferas governamentais na formação desses profissionais resulta em uma formação profissional precária. As formações geralmente ocorrem por meio de cursos oferecidos durante a graduação e mediante formações continuadas em especializações, dificultando a expansão e a segurança na execução do PDP. Isso contribui para a carência de profissionais capacitados para realizar o PDP efetivamente.

### Limitações do estudo

O estudo teve como limitação a não generalização dos resultados, inerente que as entrevistas realizadas foram com médicos obstetras de uma unidade hospitalar na região Norte do Brasil, constituindo uma realidade local que apresenta questões culturais e especificidades diferentes quanto à temática. Além disso, houve a dificuldade quanto ao tempo de realização das entrevistas, pois todos as entrevistas ocorreram no período de trabalho.

### Contribuições para as áreas da enfermagem, saúde ou políticas públicas

O conhecimento dos significados atribuídos por médicos obstetras sobre o parto domiciliar é algo fundamental para se promover discussões em prol da autonomia da mulher e também para outros profissionais não médicos, como a enfermagem obstétrica de garantia aos seus direitos na sua atuação profissional. Em muitos momentos, há na classe médica perspectivas que são contrárias à sua autonomia e direito instituído, mas é preciso que haja mudanças no modelo hegemônico obstétrico no Brasil.

Dessa forma, o estudo vem a fortalecer o direito das mulheres ao parto normal e à garantia da enfermagem obstétrica na sua atuação profissional, tendo em vista o posicionamento contrário desta prática, especialmente pela medicina e seu conselho profissional.

## CONSIDERAÇÕES FINAIS

Compreende-se que, pelos significados atribuídos pelos médicos obstetras, o parto domiciliar constitui um fator de risco, especialmente pela falta de estrutura e suporte no sistema de saúde brasileiro. Assim, mostra-se a necessidade de repensar, em detrimento dos achados científicos do tema, e fornecer um apoio de forma global, enquanto política de saúde, para a sua efetivação, conforme a equidade para o parto domiciliar.

O PDP pode ser uma opção segura para mulheres com gestações de risco habitual, caso sejam garantidas condições adequadas de infraestrutura e suporte profissional. No entanto, a segurança do PDP depende crucialmente da disponibilidade de suporte emergencial e de uma rede de saúde integrada que permita transferências rápidas para hospitais, quando necessário.

No contexto brasileiro, a viabilidade do PDP é limitada por desafios significativos, incluindo a falta de infraestrutura, a ausência de políticas públicas de suporte e a restrição econômica, que exclui a maioria das mulheres dependentes do SUS. Além disso, há um forte preconceito social e institucional contra o PDP, desestimulando a escolha por essa modalidade de parto.

Para superar essas barreiras, portanto, é necessário investir na formação e capacitação de profissionais de saúde, desenvolver políticas públicas que integrem o PDP como uma opção segura e viável, e aumentar a conscientização sobre os benefícios e riscos dessa prática. Garantir que as mulheres tenham acesso à informação desde o início da gestação é fundamental para poderem fazer escolhas informadas sobre o local e a modalidade do parto. Com um sistema de saúde mais inclusivo e preparado, o PDP pode se tornar uma alternativa segura e desejável para muitas mulheres, respeitando suas preferências e necessidades.

## Data Availability

Os dados de pesquisa só estão disponíveis mediante solicitação.
